# Phosphate–Induced Renal Fibrosis Requires the Prolyl Isomerase Pin1

**DOI:** 10.1371/journal.pone.0150093

**Published:** 2016-02-25

**Authors:** Zhong-Jian Shen, Jie Hu, Kazuhiro Shiizaki, Makoto Kuro-o, James S. Malter

**Affiliations:** Department of Pathology, University of Texas Southwestern Medical Center, Dallas, Texas, United States of America; UCL Institute of Child Health, UNITED KINGDOM

## Abstract

Tubulo-interstitial fibrosis is a common, destructive endpoint for a variety of kidney diseases. Fibrosis is well correlated with the loss of kidney function in both humans and rodents. The identification of modulators of fibrosis could provide novel therapeutic approaches to reducing disease progression or severity. Here, we show that the peptidyl-prolyl isomerase Pin1 is an important molecular contributor that facilitates renal fibrosis in a well-characterized animal model. While wild-type mice fed a high phosphate diet (HPD) for 8–12 weeks developed calcium deposition, macrophage infiltration and extracellular matrix (ECM) accumulation in the kidney interstitium, Pin1 null mice showed significantly less pathology. The serum Pi in both WT and KO mice were significantly increased by the HPD, but the serum Ca was slightly decreased in KO compared to WT. In addition, both WT and KO HPD mice had less weight gain but exhibited normal organ mass (kidney, lung, spleen, liver and heart). Unexpectedly, renal function was not initially impaired in either genotype irrespective of the HPD. Our results suggest that diet containing high Pi induces rapid renal fibrosis before a significant impact on renal function and that Pin1 plays an important role in the fibrotic process.

## Introduction

ECM homeostasis plays a critical role in the maintenance of normal renal function. This entails a delicate balance between the production and degradation of ECM, likely coordinated by interstitial fibroblasts [1–3]. During physiologic repair or pathologic processes, these cells are activated and undergo phenotypic changes to become myofibroblasts, which is likely a critical event in the initiation and progression of renal fibrosis. Along with fibroblasts, tubular epithelial cells, circulating fibrocytes, vascular pericytes, endothelial cells and glomerular podocytes can also undergo phenotypic changes and contribute to the myofibroblast pool in kidney during stress. Increased fibroblast and myofibroblast activation markers (vimentin and α-SMA) correlate with elevated serum creatinine, renal fibrosis and functional failure of the organ.

Myofibroblast transdifferentiation, proliferation and ECM production are regulated by a number of local and circulating factors, environmental stimuli, and ageing [4, 5]. These include paracrine or autocrine cytokines, direct interaction with leukocytes/macrophages, hormones, hyperglycemia and hypoxia. Of these, TGF-β is potentially the most important profibrotic cytokine responsible for myofibroblast activation and ECM production. TGF-β is synthesized ubiquitously throughout the kidney but in chronic kidney disease induced by hypoxia, inflammation, proteinuria or oxidative stress, is produced predominantly by epithelial cells [6–11].

Previous studies in animals and humans have demonstrated the connection between excess dietary phosphate, elevated plasma phosphate levels and the development of mononuclear cell infiltration, renal fibrosis and calcification [12–16]. Increasing evidence suggest that excess, extracellular phosphate has a wide range of effects on cellular physiology [17], apoptosis [18], cellular senescence [19] and oxidative stress [20], and can induce the production of ECM and pro-fibrotic molecules by fibroblasts [12, 21].

Recently, it has been shown that the peptidyl-prolyl isomerase, Pin1 plays an important role in the regulation of organ fibrosis after injury [22–24]. Pin1 was originally implicated in cell proliferation in part through control of cyclin D1 levels [25]. Pin1 specifically binds to and isomerizes pSer/pThr-Pro residues, altering target protein conformation, location, levels and/or function. We, and others have shown that Pin1 regulates cytokine production and signaling in immune cells, fibroblasts and osteoblasts [22, 23, 26]. Pin1 blockade *in vivo* significantly reduced airway inflammation and pulmonary collagen deposition in animal models of asthma and lung transplantation. Moreover, Pin1 was required for the expression of osteoblast specific transcription factors and ECM and the formation of bone nodules [26]. However, the role of Pin1 in the chronic kidney diseases is unknown. In this study, we present new evidence that Pin1 modulates renal ECM remodeling, macrophage mobilization and calcium homeostasis during HPD-induced fibrotic response. Our results suggest that Pin1 blockade can attenuate the pro-fibrogenic milieu and reduce ECM deposition during pathological renal fibrosis.

## Materials and Methods

### Materials

Anti-AKT (pan), anti-p-AKT (S473), anti-p44/42 MAPK, and anti-p-p44/42 MAPK (pTEpY) were purchased from Cell Signaling. Anti-Smad3 and anti-p-Smad3 (S423/425) were from Abcam. Monoclonal anti-β-actin was from Sigma. Anti-F4/80 and SYBR Green PCR Master Mix were from Bio-Rad. PCR primers (Table 1) were designed with Primer Express software and purchased from IDT, Inc. Protease Inhibitor Mixture was from Calbiochem. The DyLight 800/680 secondary antibodies were purchased from Thermo Scientific.

**Table 1:** qPCR primers used in this study.

Target	Forward (5'—>3')	Reverse (5'—>3')
CD68	CCAATTCAGGGTGGAAGAAA	CTCGGGCTCTGATGTAGGTC
COL1A1	ACAGACGAACAACCCAAACT	GGTTTTTGGTCACGTTCAGT
COL3A1	AACACGCAAGGCTGTGAGACT	GCCAACGTCCACACCAAATT
COL5A1	CCTGACCTTCAAAAGATGTTCTGA	GGATGTGTTGAGGGTTGTTTTAAA
CTGF	AAAGTGCATCCGGACACCTAA	TGCAGCCAGAAAGCTCAAACT
FGF-1	CCCAAGTGCCCAGCAAAT	TCATGGCGTTTGTGTCCTATG
FN-1	CCTCTGCTTTCTTTTGCCATCT	CACAGCCACAGGCCATGTC
KIM-1	GGA GAT ACC TGG AGT AAT CAC ACT G	TAG CCA CGG TGC TCA CAA GG
IL1-β	AGTTGACGGACCCCAAAAGAT	GGACAGCCCAGGTCAAAGG
MMP12	TGAGGCAGGAGCTCATGGA	AGGCTTGATTCCTGGGAAGTGT
MMP2	GGACCCCGGTTTCCCTAA	CAGGTTATCAGGGATGGCATTC
MMP3	CCCCTGATGTCCTCGTGGTA	GCACATTGGTGATGTCTCAGGTT
MMP8	CACGCACCCTATGAGGACAA	GCAGGACACGTGGGATGAGT
MMP9	GACCTTCACCCGCGTGTAC	GCTCCGCGACACCAAACT
NLRP3	TGCTCTTCACTGCTATCAAGCCCT	ACAAGCCTTTGCTCCAGACCCTAT
PAI-1	TCTCCAATTACTGGGTGAGTCAGA	GCAGCCGGAAATGACACAT
PDGFB	TTTGGAGACTTGGGCTTGGA	ACGGACCCCCAGATCAGAA
TGF-β1	GTGCTAATGGTGGACCGCA	GATGTCTTTGGTTTTCTCATAGATGG
TIMP1	ACACCCCAGTCATGGAAAGC	TCACTGCGGTTCTGGGACTT
TIMP2	TTTTGCAATGCAGACGTAGTGA	CCGGAATCCACCTCCTTCTC
TIMP3	TGCATGTCAGAGGGAGAGAAGA	GTATCATAGGGCTGGGTAACTGTGA
TIMP4	CATCCATCTGTGCAACTACATTGA	GGCTCTCCCTCTGCACCAA
TNF-α	ATG AGA AGT TCC CAA ATG GCC T	TCC ACT TGG TGG TTT GCT ACG
18S	CATTAAATCAGTTATGGTTCCTTTGG	TCGGCATGTATTAGCTCTAGAATTAC

### Pin1^−/−^ mice

Pin1^+/−^ mice on a pure C57BL/6J background were obtained from T. Means (Duke University, Durham, North Carolina, USA) [22]. Pin1^-/-^ (KO) mice were produced by crossing heterozygotes. Animal care was carried out in strict accordance with the recommendations in the Guide for the Care and Use of Laboratory Animals of the National Institutes of Health. Our protocol was approved by the Committee on the Ethics of Animal Experiments of the University of Texas Southwestern Medical Center (Permit Number: 2011–0139). All surgery was performed under sodium pentobarbital anesthesia, and all efforts were made to minimize suffering. Mixed gender (balanced in numbers) were used for all experiments unless otherwise indicated.

### Induction of renal fibrosis with high phosphate diet (HPD)

Mice (12-week old, 6 mice/group) were fed normal food (controls) (0.35% inorganic phosphate/Pi) or high Pi (2.0%) diet [16, 27–30] for 4, 8 and 12 weeks. Body weight in each group was measured at the beginning and end of the diet. At the end, mice were euthanized, and blood and organs (kidney, lung, spleen, liver and heart) were harvested. Serum biochemistry, histology and inflammation were evaluated by the method described below.

### Reverse transcription and real-time PCR

Whole kidney RNA was extracted with TriReagent. cDNA Quantitative PCR was performed with a SYBR PCR master mix with the primers shown (Table 1). An ABI 7500 thermocycler (Applied Biosystems, Foster City, CA) was used for 45 cycles of PCR. ΔCT calculates the differences between target CT values and the normalizer (housekeeping gene) for each sample: ΔCT = CT (target) − CT (normalizer). The comparative ΔΔCT calculates the differences between each sample ΔCT value and the baseline ΔCT. The comparative expression level (fold changes) was obtained transforming the logarithmic values to absolute values using 2^−ΔΔCT^. All data from untreated control cells was normalized to fold change "1".

### Histological assessment of kidney collagen

Paraffin sections of the kidney tissue were prepared and stained using a modified trichrome stain with Alcian blue as described previously [31]. The intensity of trichrome staining in outer medulla was quantified using ImageJ software (http://rsb.info.nih.gov/ij/). Results are expressed as the intensity of trichrome staining per field (20x). At least 3 fields /slide and 6 mice/group were measured for statistics.

### Immunostaining and immunoblots

For the analysis of macrophage infiltration, tissue sections were permeabilized, blocked, and incubated with appropriate dilutions of primary anti-mouse F4/80 (Bio-Rad) followed by HRP-conjugated secondary antibodies and DAB substrate. Images were collected by light microscopy and brown color intensity in outer medulla was quantified using Photoshop and ImageJ. Results are expressed as the intensity of the color per field (20x). At least 3 fields/slide and 6 mice/group were analyzed. For immunoblots, whole kidney lysates were prepared in Nonidet P-40 buffer and protein concentration was measured prior to immunoblots. The proteins were transferred onto nitrocellulose membranes and probed with primary and secondary antibodies. Protein bands were detected and quantified using LI-COR^®^ Odyssey^®^ Imaging System.

### Serum biochemistry and von Kossa staining

The serum calcium, K, Vitamin D, BUN, CRE and phosphate (Pi) concentrations were measured on an Ortho Clinical Vitros 250 Chemistry System in the Mouse Metabolic Phenotyping Core at UT-Southwestern Medical Center, and active form of Vitamin D3 by ELISA. Von Kossa staining for calcium-phosphate was performed at Molecular Pathology Core. The percentage of positive area (dark color) in outer medulla was quantified as shown in Figures. At least 3 fields/slide and 6 mice/group were analyzed.

### Statistical Analyses

Statistical evaluations of the data were conducted by using paired Student’s t test for per-comparison analysis. *p*<0.05 was considered statistically significant. The data were presented as mean ± S.D.

## Results

### Serum biochemistries and renal function after HPD

**Fig 1:**
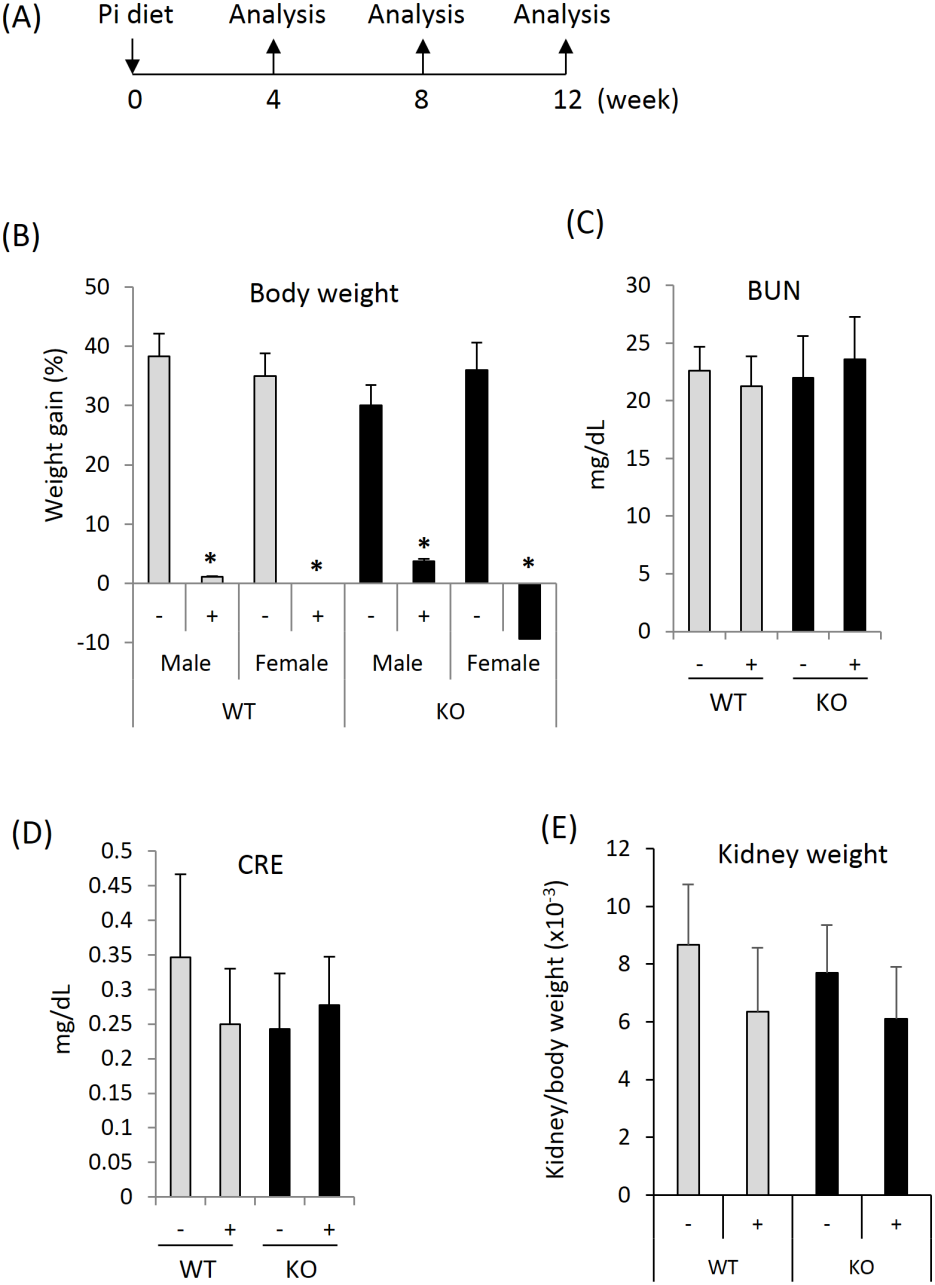
Organ and body weight and serum biochemistries after HPD. (A) 12-week old WT or Pin1 KO mice were fed HPD for 4, 8, 12 weeks before analysis. (B) Mouse body weights were measured at the beginning and end of normal diet (-) and HPD (+), and expressed as % increase compared to the weight at the beginning of diet given. * denotes p<0.01 between treatments with 6 mice/group. (C) and (D) Serum from each mouse was collected at the end of diet (12 weeks) and measured for the indicators of kidney function. (E) Kidney weight at the end of diet.

WT and KO animals were fed two different diets that contained identical amounts of protein, fat, carbohydrate, basic mineral and vitamins but differed in the amount of inorganic Pi (0.35% in normal diet and 2% in HPD) present. Mice were started on HPD or control diets at 12 weeks of age prior to harvest after 4, 8 and 12 weeks (Fig 1A). After fixation, the kidneys were stained with H&E and Masson's trichrome.

**Fig 2:**
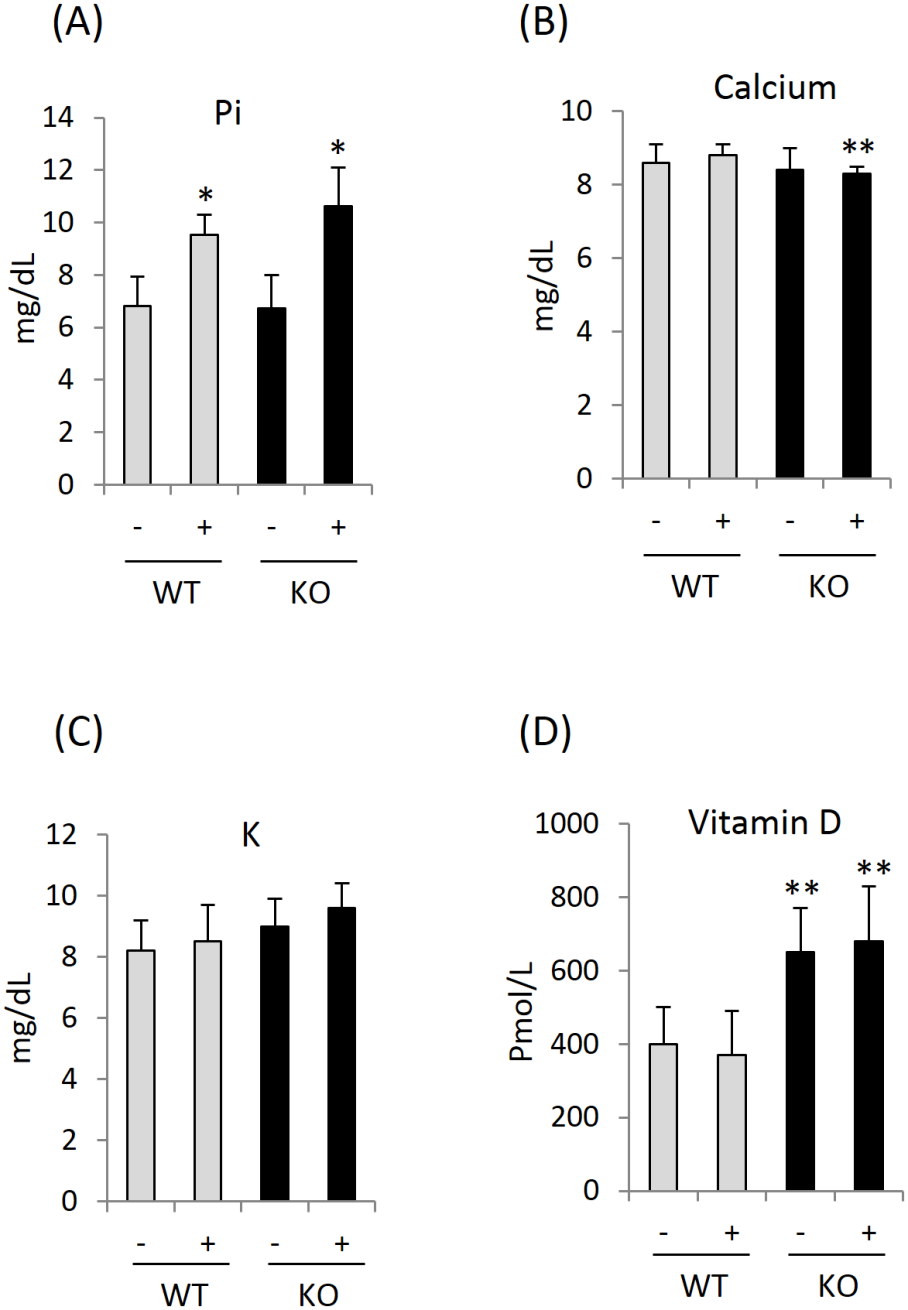
Serum electrolytes. Serum from each mouse were collected at the end of feeding (12 weeks) and measured for the electrolyte concentration (A) (B) (C) and the amount of Vitamin D (D). ** in (B) and (D) denotes p<0.05 between genotypes with (+) or without (-) HPD. * in (A) denotes p<0.05 between treatments (+ vs. -) in each genotype. All data were analyzed by student t-test with 6 mice each group.

We weighed mice in each group the day before and 4, 8, and 12 weeks after initiation of HPD. Although changes in kidney weight were of primary interest, the weights of the other organs were also recorded at each time points. HPD slowed the gaining of body weight and even resulted in weight loss in KO female mice (Fig 1B and data not shown for 4-week and 8-week points) compared to non-HPD female controls. Despite this, serum creatinine and BUN were unchanged between control and experimental groups (Fig 1C and 1D), suggesting adequate filtration capacity still remains. The gross appearance (size, color and shape) and mass (normalized to terminal body weight) of the organs (kidney, liver, spleen and lung) were entirely normal (Fig 1E and data not shown). HPD significantly increased serum Pi in both genotypes (Fig 2A) while Ca remained at the control level during the experimental period in WT mice (Fig 2B). Plasma Ca is often lowered by elevated phosphate due to their co-precipitation in tissues. Lower Ca levels were seen in Pin1 KO mice after HPD (Fig 2B) without significant change in potassium levels (Fig 2C). On the other hand, Pin1 deficiency was associated with increased serum vitamin D regardless of HPD (Fig 2D), which may contribute to the decreased fibrotic phenotype observed in KO mice [32].

### Renal histology after HPD

**Fig 3:**
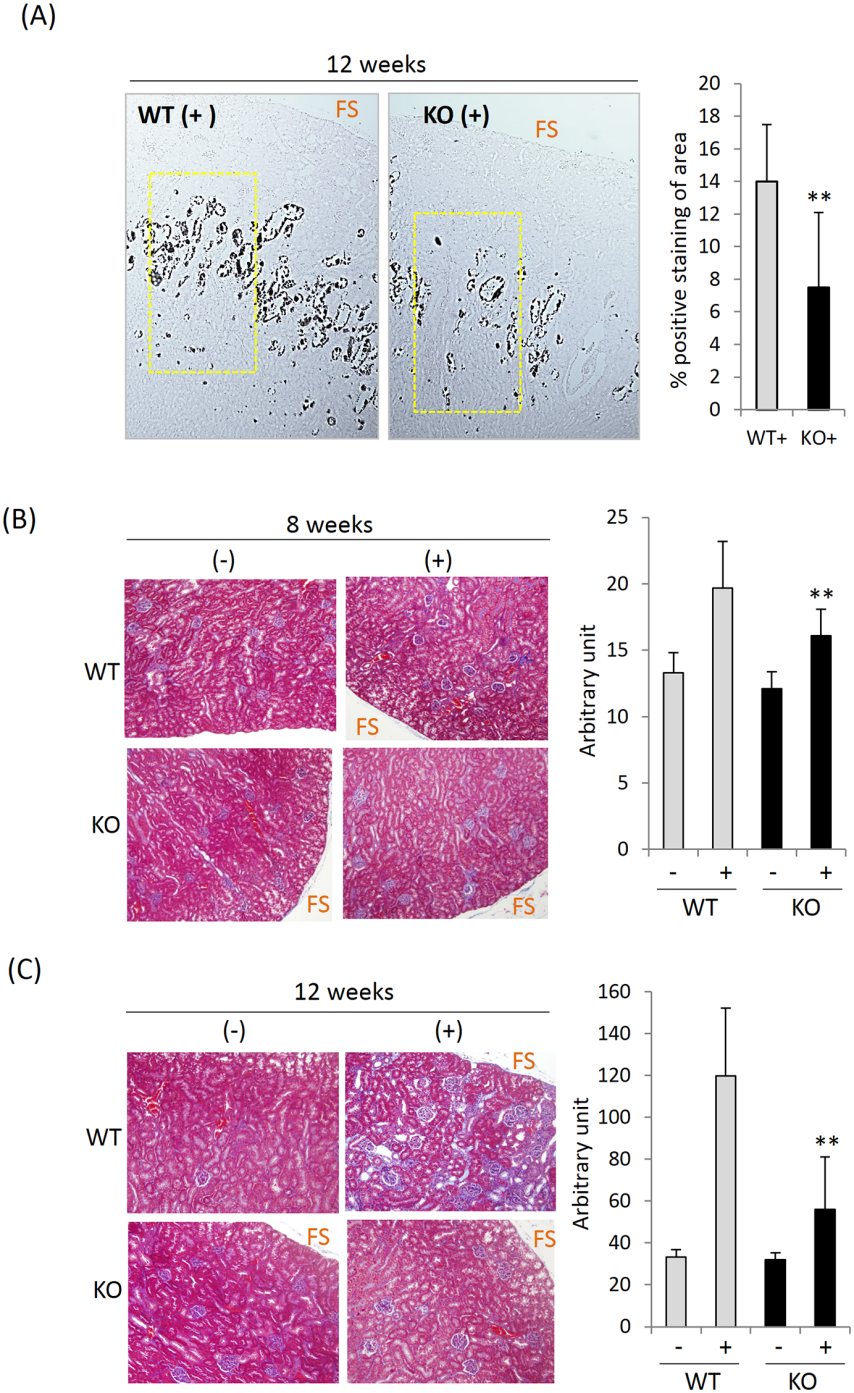
Collagen deposition by HPD is decreased by Pin1 deficiency. (A) Kidney sections from 12-week HPD (+) mice were stained with Von Kossa for calcium-phosphate containing salts (dark) and the positive area in outer medulla was quantified as shown and described in the Methods. (B) and (C) Kidney sections from 8 and 12-week control diet (-) and HPD (+) mice were stained with Masson’s trichrome (left) for total collagen deposition. The intensity of blue color was quantified and shown in bar graphs (right). FS: free surface of kidney. ** denotes p<0.05 between genotypes by student's t-test with 6 mice each group.

WT mice fed HPD for 4 weeks showed largely normal renal histology (S1 and S3 Figs). However, after 8-weeks, the kidneys showed fairly disorganized nephron loops and collecting ducts that occasionally contained casts. The cortex was also involved but to a lesser extent. At 8–12 weeks, microscopic examination revealed calcium-phosphate deposits in the outer medulla as detected by Von Kossa staining (dark areas in Fig 3A and S1 Fig). This was specific for kidney as the liver, lung, brain, fat and small intestine were free of deposits (S2 Fig and data not shown). Not surprisingly, the level of kidney injury molecule-1 (KIM-1), a biomarker of renal tubular injury, was considerably higher in HPD kidney (S4 Fig). Despite the tubular injury, cortical glomeruli remained fairly well preserved in all HPD groups and showed minimal changes. Comparably treated KO mice showed much less injury than WT mice (Fig 3A, S1 and S4 Figs). At 12 weeks, (Fig 3B and 3C, S1, S3 and S4 Figs), KO mice showed approximately 50% the collagen deposition (Fig 3C) as WT animals and barely above untreated controls. Despite the renal toxicity of the HPD, no abnormalities were found in histopathologic sections of the lung, spleen and liver (data not shown) or in fibrogenic gene expression profiles in the lung (not shown). Based on prior studies, the fibrotic lesions found in the kidney of HPD fed mice likely resulted from calcification-induced injury [13, 33]. In aggregate, these results suggest that Pin1 is required for collagen accumulation after renal injury induced by HPD.

### Pin1 is required for ECM gene expression after HPD

**Fig 4:**
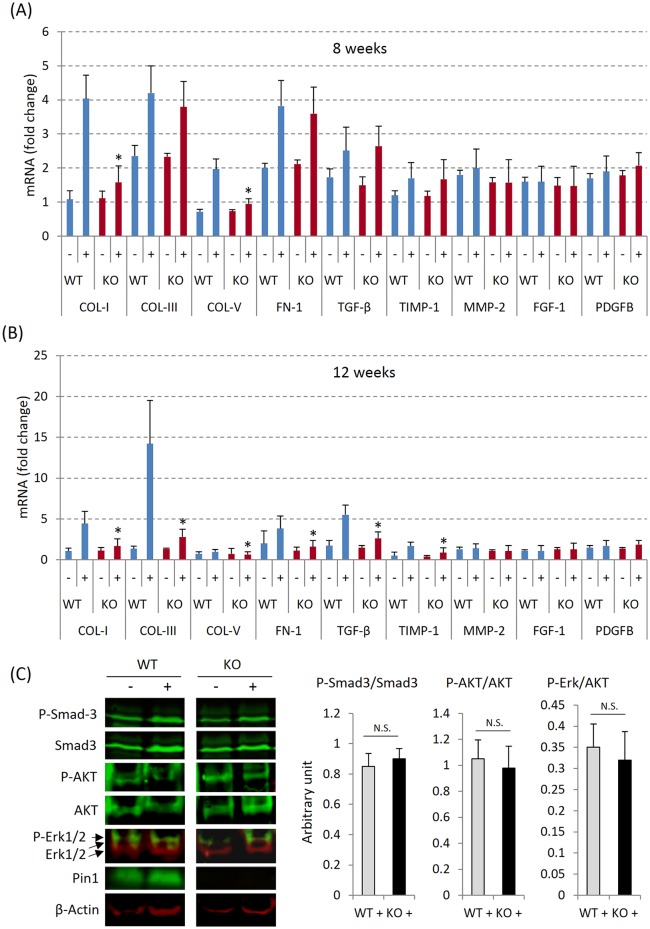
Renal Expression of ECM mRNAs. (A) and (B) Kidney RNA from 8- and 12-weeks HPD mice was subjected to quantitative PCR. * denotes p<0.05 between genotypes with HPD (+) by student's t-test with 6 mice each group. (C) Whole kidney lysates from 12-weeks control and HPD mice were subjected to immunoblots with antibodies shown. The lysates were from 3–6 animals each group. Phosphorylated proteins were normalized to total proteins, and shown in bar graph (right). N.S.—not significant.

Given the reduction in collagen deposition in KO versus WT mice, we examined the expression of individual ECM genes in whole kidney known to be involved in tissue fibrosis (primers shown in Table 1). As shown, collagens I/III/V, FN-1, TGF-β1 and TIMP-1 mRNA were significantly upregulated in WT kidney after 8–12 weeks of HPD. Some genes (Col III and TGF-β1) showed greater induction after HPD at 12 weeks than at 8 (Fig 4A and 4B). Consistent with the trichrome staining (Fig 3), compared to WT, Pin1 KO mouse kidneys showed significantly less collagen I and V mRNA accumulation after 8 weeks of HPD and significantly less mRNA of all 6 fibrosis-related genes (collagens I/III/V, FN-1, TGF-β1 and TIMP-1 mRNA) after 12 weeks. The levels of TIMP-2/3/4, MMP-2/3/8/9, CTGFB, PAI-1, FGF-1 and PDGF genes were not altered by HPD or Pin1 deficiency.

As TGF-β1 signaling is pivotal for the induction and maintenance of many forms of renal fibrosis, we examined primary signaling events by immunoblotting for phospho-Smad3. Under basal conditions, there were low but detectable amounts of phospho-Smad3 in the kidney which was not altered by HPD or Pin1 deficiency (Fig 4C) irrespective of the changes in TGF-β1 mRNA and its target genes (Fig 4A and 4B). Neither Erk1/2 MAPK nor AKT activation was changed by HPD. These data suggest that non-Smad pathways mediate TGF-β signaling or factors other than TGF-β contribute to the observed ECM production.

### Pin1 is required for renal inflammation after HPD

**Fig 5:**
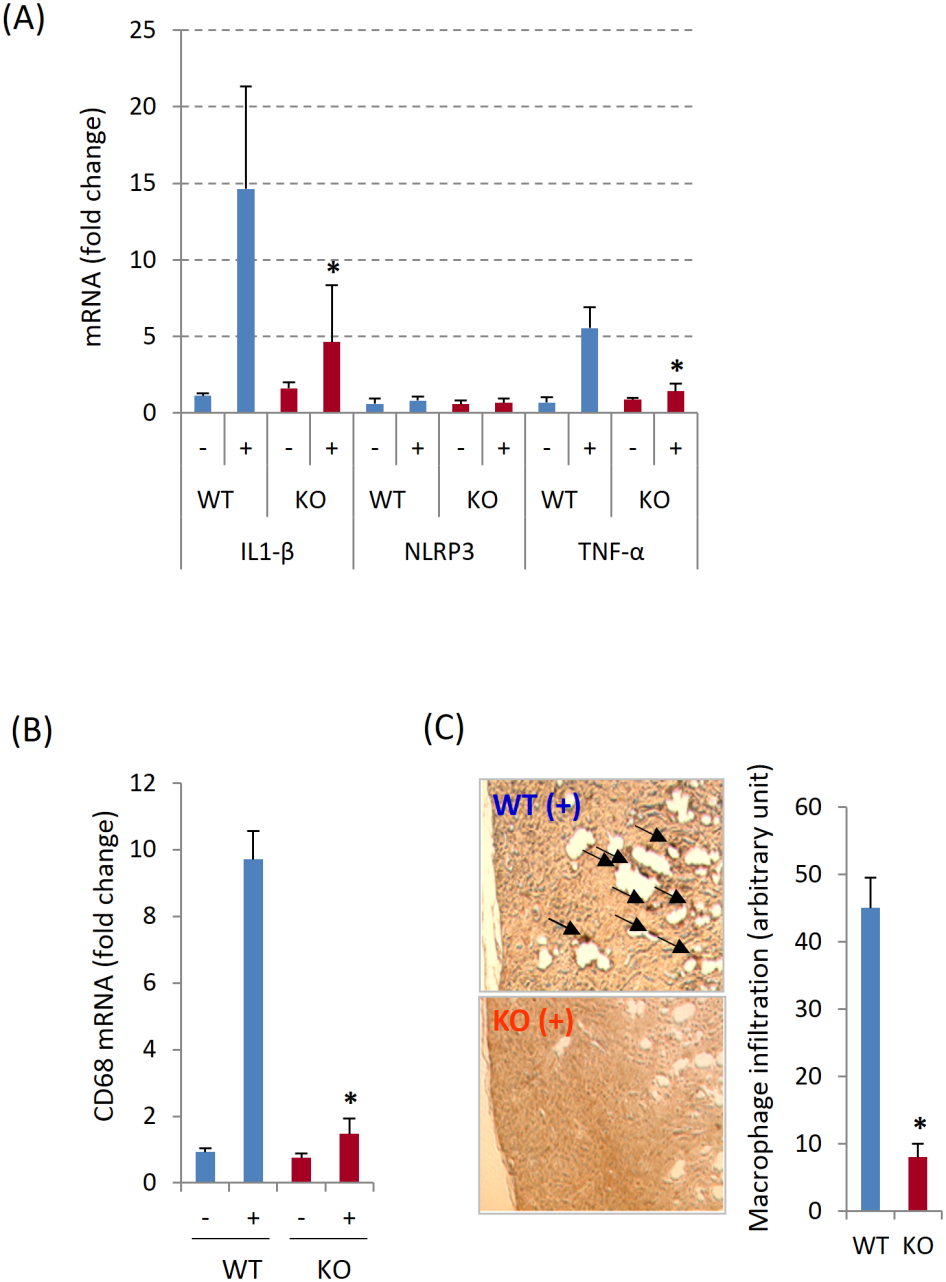
HPD-induced kidney Inflammation is attenuated by the Pin1 deficiency. (A) The expression of inflammasome component (NLRP3) and pro-inflammatory cytokines (IL-1β and TNF-α) in kidney from 12-weeks HPD (+) and control mice (-) were analyzed by quantitative PCR. (B) Macrophage infiltration was assessed by the expression of CD68. (C) Macrophages (left) were stained with anti-F4/80 and the density of brown color (arrow-heads) in the outer medulla was quantified (right) from 3 fields (20x) each slide. * denotes p<0.05 between genotypes with HPD with 6 mice per group.

Local inflammation frequently accompanies tissue fibrosis. In particular, macrophages are recognized as a key effector in many forms of renal fibrosis and ECM accumulation [34]. Therefore we analyzed pro-inflammatory cytokine expression and macrophage infiltration in the kidney from 12-weeks HPD mice as they show significant phenotypic changes (S1, S3 and S4 Figs). Based on qPCR, IL1-β and TNF-α mRNAs were significantly increased in WT mice (Fig 5A). However, while somewhat increased, both cytokines were significantly lower in HPD Pin1 KO animals than WT. Similar to the previous findings [34–36], F4/80+ macrophage infiltration (Fig 5B and 5C) into calcium deposition areas (Fig 5C) was also observed after HPD, which was almost completely blocked in the absence of Pin1. These data indicate that Pin1 contributes to the kidney inflammation (cell recruitment and cytokine expression) during HPD-induced fibrosis.

## Discussion

In this study, we have identified Pin1 as a critical regulator of renal fibrosis, calcium deposition, tubular injury and inflammation after HPD. Despite significant morphologic and molecular changes, adequate renal function was present to maintain normal serum BUN and CREA. Surprisingly, the level of renal P-Smad3 was unchanged by the HPD or Pin1 deficiency, suggesting a non-canonical signaling pathway was responsible for ECM production by damaged parenchymal cells. Pin1 null mice displayed lower plasma Ca concentrations compared to WT after HPD. In aggregate, these results suggest that Pin1 regulates HPD-induced renal fibrosis through non-Smad pathways and possibly through modulation of plasma Ca.

While elevated plasma Pi can be a consequence of chronic renal failure, in humans, Pi has also been associated with progressive renal disease independent of glomerular filtration rate (GFR) [14]. Consistent with this, animals fed HPD developed increased plasma Pi (Fig 2A) with subsequent kidney fibrosis, connective tissue calcification and mononuclear cell infiltration [27–30], mirroring the findings in present study. Conversely, restricting dietary Pi prevented renal injury and failure in experimental models of kidney disease [15, 28, 37, 38]. These data strongly suggest that excessive Pi is intrinsically linked to renal diseases and that HPD is a valid and powerful approach to characterize how abnormalities in this key electrolyte cause disease.

The mechanisms underlying HPD mediated kidney damage are likely multifaceted. Elevated plasma Pi (Fig 2A) may directly damage the microvascular endothelium within the kidney leading to ischemia/hypoxia [39–41] with consequent fibrosis. Hyperphosphatemia directly affects gene expression in epithelial and mesenchymal cells (e.g. fibroblasts), increasing the production of ECM and pro-fibrotic proteins [12, 42]. Finally, high circulatory Pi induces the formation and deposition of calcium-phosphate microcrystals in the tubular lumen, peritubular space, capillaries and interstitium that likely activates inflammatory and fibrotic responses and induces tubular atrophy [43–46], despite normal levels of serum Ca (Fig 2B). Early dietary restriction of Pi preserved residual renal function, delayed the progression of renal injury and was associated with reductions in calcium-phosphate crystals [15, 47]. However, in cats with spontaneous CRF, elevated plasma Pi was associated with interstitial fibrosis but not tubular mineralization [48].

At a molecular level, ECM production is commonly mediated by Smad activation. Unexpectedly, Smad3 phosphorylation (Fig 4C) was unchanged after HPD in either WT or Pin1 KO mice despite the activation of ECM genes and the presence of fibrosis (after 8–12 weeks). These data suggest that non-canonical signaling pathways, independent of Smads but still subject to Pin1 regulation are triggered after HPD. Based on prior data, the most likely alternative pathway involves ERK and PI3K/Akt [49]. In MEF and hepatic stellate cells, TGF-β1 and α-SMA transcription were dependent on Pin1 that was controlled by Erk MAPK and PI3K/Akt and downstream AP-1 [50]. However, we did not see significant changes in the activation of these signaling molecules in the present study (Fig 4C), suggesting other non-Smad pathways modulate the observed phenotypes altered by HPD or Pin1 deficiency.

Pin1 regulates diverse cellular process including growth factor and hormone signaling, cell-cycle progression, and stress responses [25]. A role in mineral metabolism has recently been shown as well. After HPD, both Pin1 WT and KO mice showed elevated serum Pi whereas KO mice (12-week old) had lower serum calcium. Under normal diet, the level of serum calcium was normal in KO mice [26]. The hypocalcemia, however, seen in HPD-fed KO mice may be protective as it should reduce co-precipitation with Pi in kidney and other tissues. Adult mice lacking Pin1 are physically healthy but exhibit transiently elevated serum Pi, vitamin D3 and PTH and lower serum ALP activity [25, 26, 51]. Presumably the electrolyte changes induced by HPD are partially offset by the endocrine alterations induced by Pin1 deficiency.

In summary, serum Pi driven by chronic renal disease or elevated by excess dietary Pi drives or initiates renal injury and triggers a Pin1 dependent, ECM remodeling process. These results suggest that Pin1 blockade combined with Pi restriction could be an effective therapeutic option for chronic kidney diseases and renal failures.

## Supporting Information

S1 Fig Deposition of calcium-phosphate in kidney of Pin1 mice (4, 8 and 12 weeks after HPD). Kidney sections from three time points were stained with Von Kossa and imaged at 2.5x to show the extent of damage. Representative images are shown from 4–6 mice each group.
(TIF)

S2 Fig Von Kossa staining for calcium-phosphate deposits in other tissues. Sections of small intestines **(A**), liver **(B)** and fat **(C)** from 12-weeks HPD (+) and control (-) mice were stained with Von Kossa stain and imaged at 2.5x. Representative images are shown from 4–6 mice each group.
(TIF)

S3 Fig Collagen deposition in the kidney of Pin1 KO mice (4, 8 and 12 weeks after HPD). Kidney sections from three time points were stained with Masson's trichrome and imaged at 2.5x to show the extent of fibrosis. Representative images are shown from 4–6 mice each group.
(TIF)

S4 Fig HPD induces renal tubular injury, which is attenuated in the absence of Pin1. **(A-C)** Total RNA from kidney was subjected to RT-qPCR for the expression of kidney injury marker-1 (KIM-1). * denotes p<0.05 between treatments (- v.s. +) while ** denotes p<0.05 between genotypes (KO v.s. WT) with 4–6 mice/group. No significant differences were found in 4-weeks mice **(C)**.
(TIF)
