# Supplementary figures and images for: Phosphate–Induced Renal Fibrosis Requires the Prolyl Isomerase Pin1
Source: PLoS One. 2016 Feb 25;11(2):e0150093. doi: 10.1371/journal.pone.0150093 (PMC4767802; doi:10.1371/journal.pone.0150093)

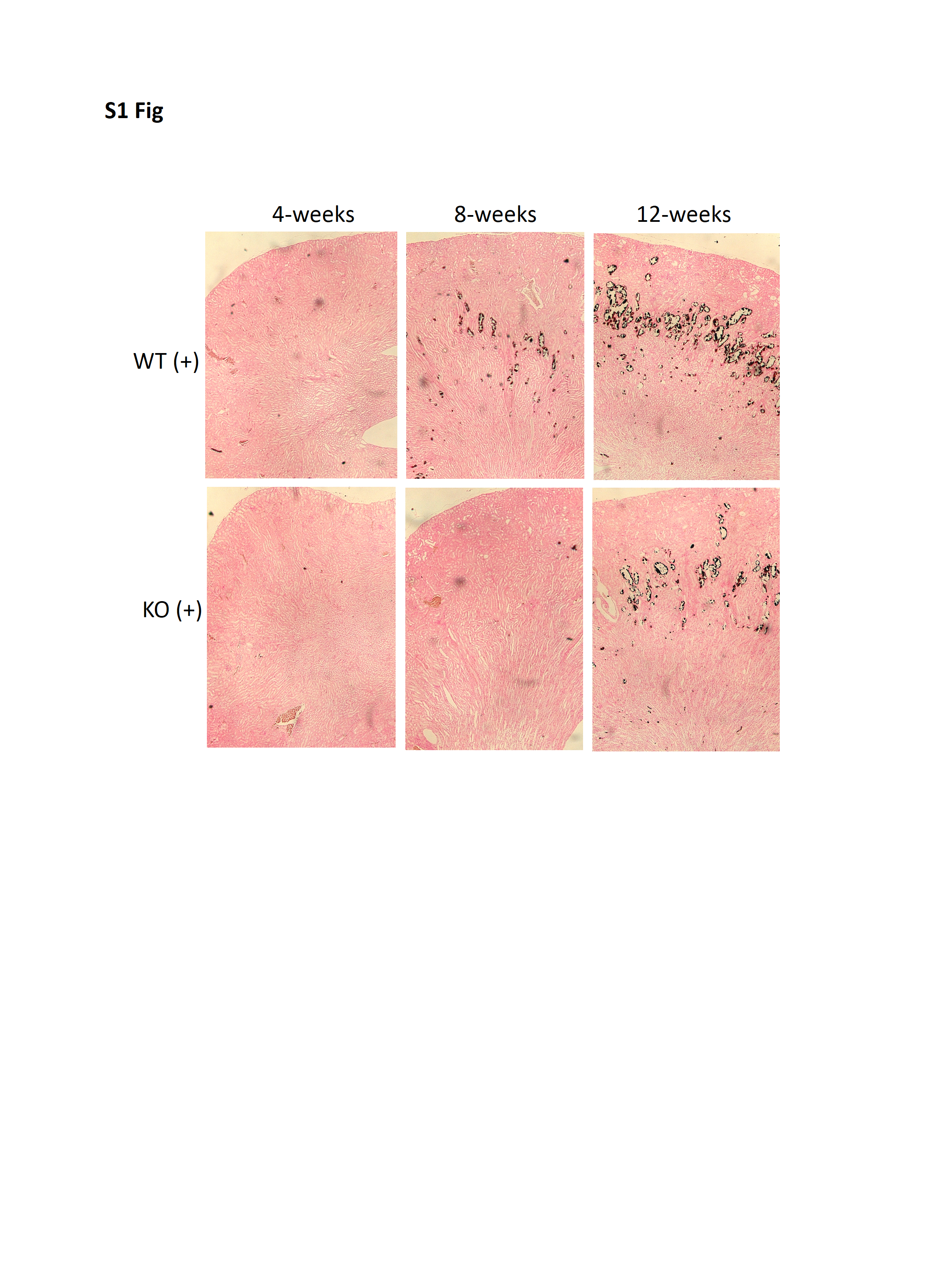

Supplement: S1 Fig — Kidney sections from three time points were stained with Von Kossa and imaged at 2.5x to show the extent of damage. Representative images are shown from 4–6 mice each group. (TIF) [file pone.0150093.s001.TIF]

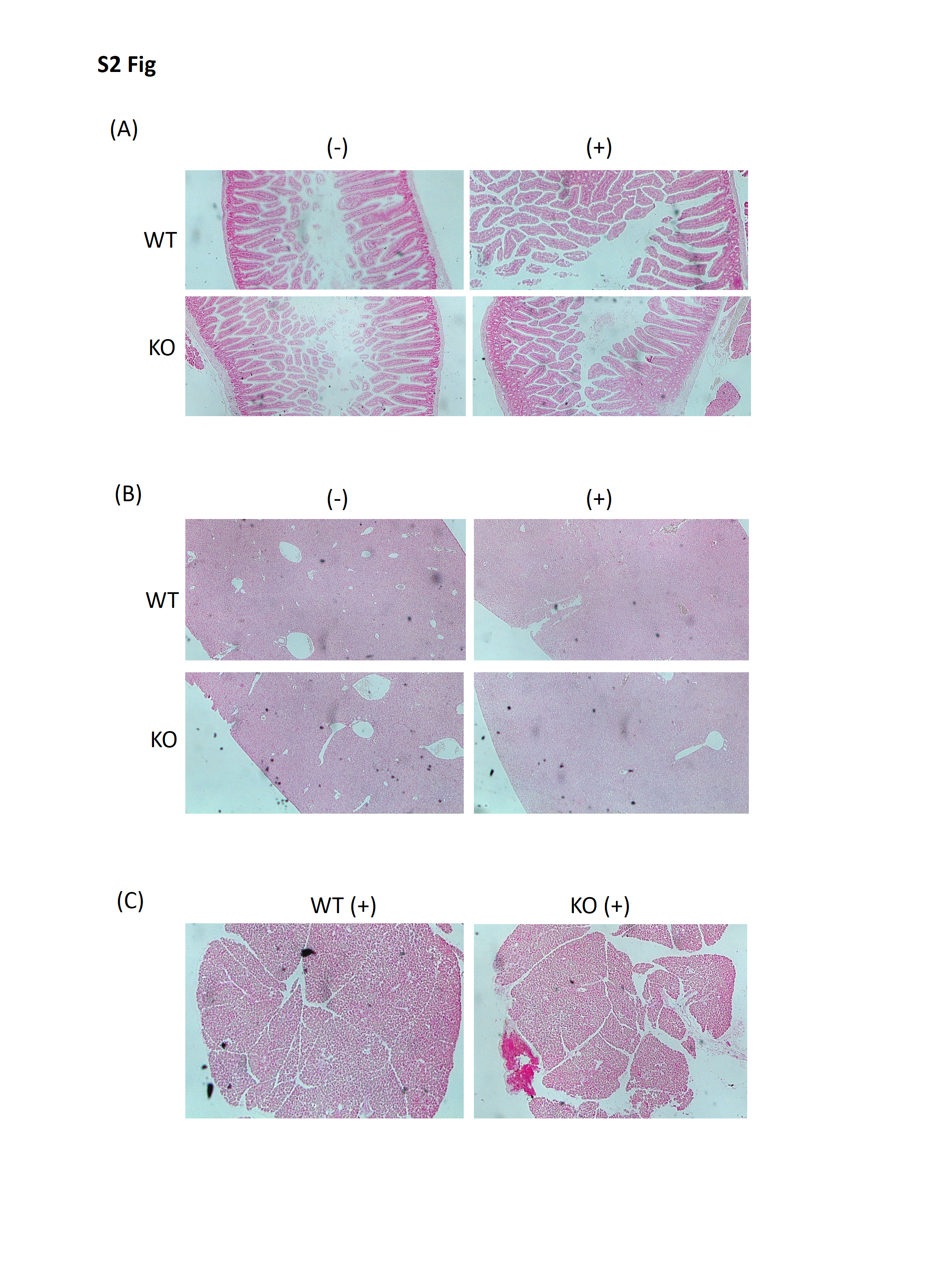

Supplement: S2 Fig — Sections of small intestines (A), liver (B) and fat (C) from 12-weeks HPD (+) and control (-) mice were stained with Von Kossa stain and imaged at 2.5x. Representative images are shown from 4–6 mice each group. (TIF) [file pone.0150093.s002.TIF]

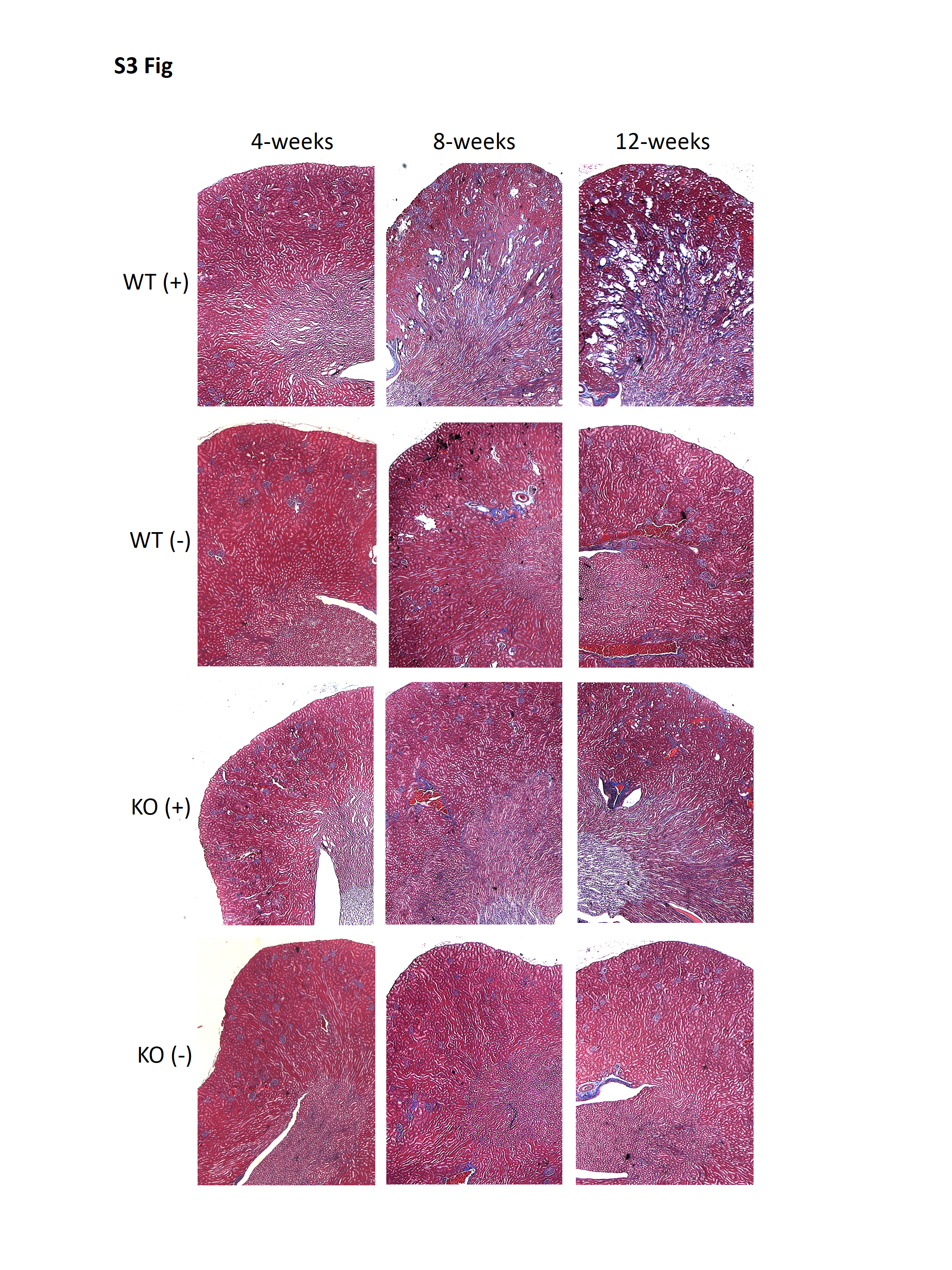

Supplement: S3 Fig — Kidney sections from three time points were stained with Masson's trichrome and imaged at 2.5x to show the extent of fibrosis. Representative images are shown from 4–6 mice each group. (TIF) [file pone.0150093.s003.TIF]

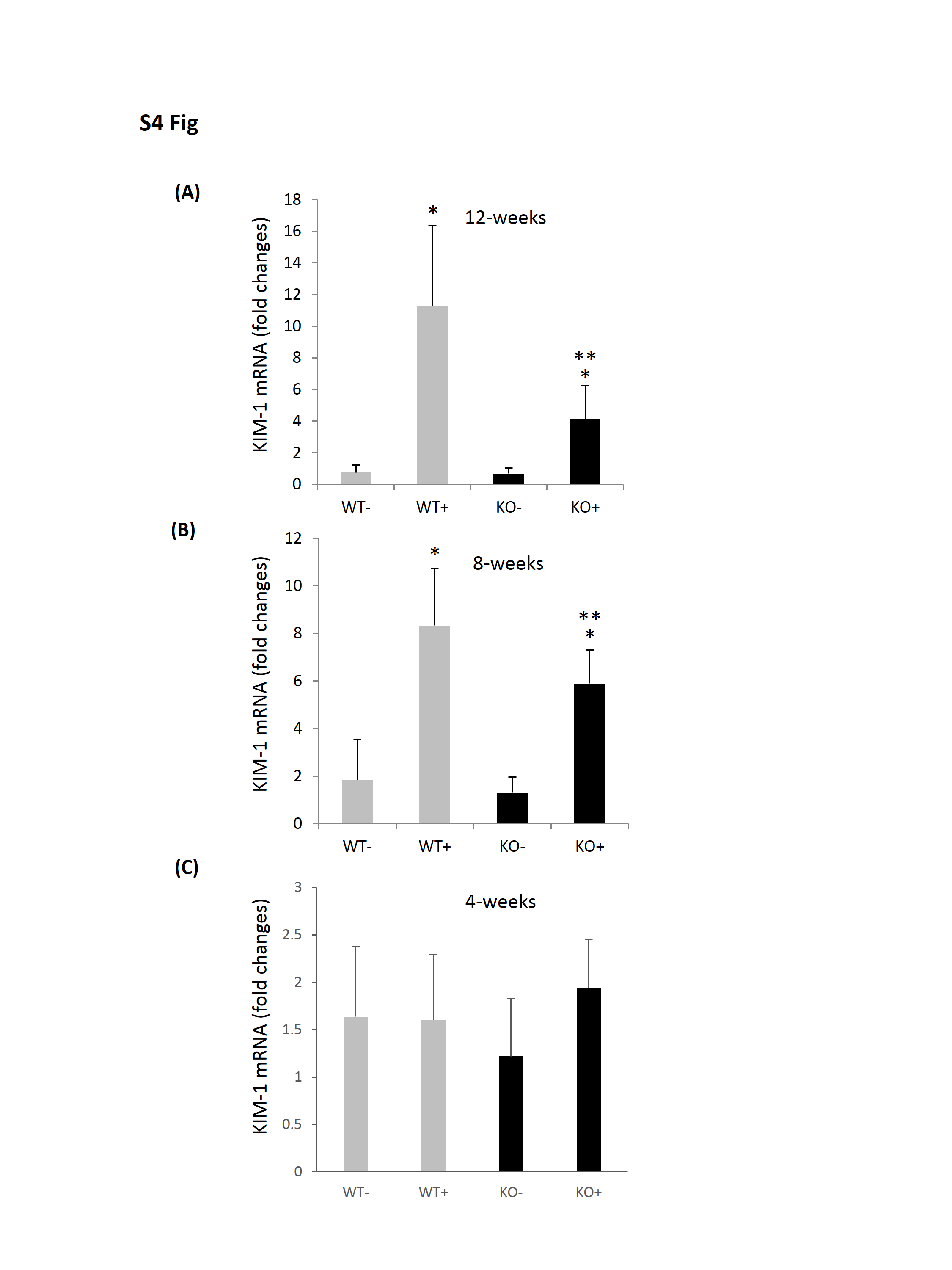

Supplement: S4 Fig — (A-C) Total RNA from kidney was subjected to RT-qPCR for the expression of kidney injury marker-1 (KIM-1). * denotes p<0.05 between treatments (- v.s. +) while ** denotes p<0.05 between genotypes (KO v.s. WT) with 4–6 mice/group. No significant differences were found in 4-weeks mice (C). (TIF) [file pone.0150093.s004.TIF]
